# 

**Published:** 1889-01

**Authors:** 


					﻿Vol. IX.
JANUARY, 1889.
NO. 1?
THE
HOMEOPATHIC pHY£lClA]fl,
A Monthly Journal of Homoeopathic Materia Medica
and Clinical Medicine.
"He is a freeman whom truth makes free, and alt are slaves beside."
’ "Seek the Truth: come whence it may, cost what it will."
EDITED AND PUBLISHED BY
Edmund j.	Nf, ix;
AND
WALTER M. JAMES, M. D.
PHILADELPHIA :
No. 1123 SFRTT<3E STREET?
Tj O 2ST T3 O W •
ALFRED HEATH A CO., Sole Agents bob Great Britain,
114 Ebury Street, Eaton Square.
- Yearly Subscription, $2.SO. invariably in advance. Single numbers, 25 cts.
Entered at the Philadelphia Poet-Office as Second-Close Mall Matter.
				

